# Application of Cre-lox gene switch to limit the Cry expression in rice green tissues

**DOI:** 10.1038/s41598-017-14679-0

**Published:** 2017-11-06

**Authors:** Hao Chen, Ju Luo, Peng Zheng, Xiaobo Zhang, Cuicui Zhang, Xinyuan Li, Mugui Wang, Yuqing Huang, Xuejiao Liu, Mehmood Jan, Yujun Liu, Peisong Hu, Jumin Tu

**Affiliations:** 1 Institute of Crop Science, College of Agriculture and Biotechnology, Zhejiang University, Yu-Hang-Tang Road No 866, Hangzhou, 310058 China; 2 State Key Laboratory of Rice Biology, China National Rice Research Institute. Ti-Yu-Chang Road No 359, Hangzhou, 310006 China

## Abstract

The presence of genetically modified (GM) protein in the endosperm is important information for the public when considering the biological safety of transgenic rice. To limit the expression of GM proteins to rice green tissues, we developed a modified Cre-lox gene switch using two cassettes named KEY and LOCK. KEY contains a nuclear-localized Cre recombinase driven by the green-tissue-specific promoter *rbcS*. LOCK contains a *Nos* terminator (*NosT*), which is used to block the expression of the gene of interest (GOI), bounded by two *loxP* sites. When KEY and LOCK are pyramided into hybrid rice, a complete gene switch system is formed. The Cre recombinase from KEY excises *loxP*-*NosT* in LOCK and unlocks the GOI in green tissues but keeps it locked in the endosperm. This regulatory effect was demonstrated by *eYFP* and *Bt* expression assays. The presence of *eYFP* and *Cre* were confirmed in the leaf, sheath, stem, and glume but not in the root, anther or seed of the gene-switch-controlled *eYFP* hybrids. Meanwhile, gene switch-controlled Bt hybrid rice not only confined the expression of Bt protein to the green tissues but also showed high resistance to striped stem borers and leaffolders.

## Introduction

The global cultivation area of genetically modified (GM) plants, including corn, canola, soybean, and cotton, has increased consistently since their commercialization in 1996, reaching 185.1 million hectares in 2016^1^. In contrast, the commercial release of transgenic rice has not yet been approved^2^. This situation has continued even after the elite transgenic insect-resistant rice T51-1 and its derivative hybrid Shanyou63/Bt^3^ passed biosafety assessment and were awarded a biosafety certification by the Chinese government in 2009^4^ that was renewed in 2014^2^. One possible reason is that as the first generation of transgenic technology product, T51-1 and its derivative hybrid Shanyou63/Bt, containing Bt driven by the constitutively active rice *Actin I* promoter, were endosperm-expressed and thus carried an insecticidal protein in their edible parts. Thus, the public may be concerned about whether GM food is safe^5^.

To relieve the concerns of the public regarding GM food biosafety, promoters specific to green tissues have been intensively studied to reduce the expression of transgenes in harvestable tissues. To date, numerous photosynthesis-related gene promoters, such as *PEPC* (phosphoenolpyruvate carboxylase)^6^, *rbcS* (ribulose-bisphosphate carboxylase)^7^, *Pdk* (pyruvate orthophosphate dikinase)^8^, *LHCPII* (light-harvesting chlorophyll a ⁄ b binding protein of photosystem II)^9^, *LP2* (the rice Leaf Panicle 2 gene)^10^, *D54O* (photosystem II 10 kDa polypeptide)^11^ and *PNZIP* (*Pharbitis nil* leucine zipper)^12^, have been shown to activate expression in green tissues and minimize the presence of gene of interest (GOI) products in seeds. However, these promoters do not provide completely tissue-specific expression of the GOI due to background expression in the seed. For instance, the amount of GM protein detected in *rbcS*-driven Cry1C rice endosperm was 2.6 ng g^−1^ in the tested samples^7^ and that in *PEPC*-driven Cry1Ab maize kernels was 15–18 ng mg^−1^ soluble protein^6^. A trace amount of transgene product was also observed in *PNZIP*-driven Bt-cotton seed^12^ as well as in *PDX1*-driven GUS-rice endosperm^13^.

Although there is no scientific basis or credible evidence for a food safety concern regarding Bt insecticidal proteins, developing transgenic rice that produces no GM protein in the endosperm would nonetheless be helpful^14^. Additionally, no foreign genes should be expressed in the edible parts in order to reassure the public regarding the safety of GM food^7,11^. Finally, new approaches must be developed to reduce the unnecessary burden of energy consumption on plant metabolism compared with the previously used constitutive promoters^7^. Therefore, in summation, the development of a highly efficient method of GOI expression in the leaves and stems of GM crops, which simultaneously and completely avoids expressing the GOI in the edible parts, will prove to be useful in transgenic research.

In plant genetic engineering, Cre-lox, one of the most common site-specific recombination systems, has been often used to control or manipulate exogenous gene expression^15–22^. The Cre-lox site-specific recombination system functions through interactions between Cre recombinase and its specific *loxP* recognition sites^18,20,22,23^. When cells that have *loxP* sites in their genomes express Cre, a recombination event can occur between two *loxP* sites^24,25^. In general, Cre recombinase has a 70% recombination efficiency without the aid of any accessory factors, and it can act on a variety of DNA substrate structures, including linear, circular and even supercoiled DNA^26–28^. The use of the Cre-lox system in plant genome manipulation has been demonstrated to remove unwanted DNA^20^, such as removing selectable markers from transgenic plants^29,30^; effectively reduce complex transgene insertions to single copies^31,32^; engineer chromosomes^33,34^, etc. The transcription terminator of a gene or operon halts transcription by providing signals in the newly synthesized mRNA that trigger a process that releases the mRNA from the transcriptional complex^35^. Currently, the *Agrobacterium* nopaline synthase terminator (*NosT*) is the most common generic recombinant element in plant genetic engineering^15^.

Here, we report a modified Cre-lox gene switch using two individual cassettes called KEY and LOCK. The KEY cassette contains a nuclear localization signal-tagged Cre recombinase driven by the green-tissue-specific promoter *rbcS*. The LOCK cassette contains a *Nos* terminator, which can block the expression of the GOI, bounded by two *loxP* sites. Our results demonstrate, based on an examination of eYFP protein and Bt application, that the Cre-lox gene switch formed when these two cassettes are combined can strictly confine the expression of the GOI to the green tissues, such that no accumulation of GM protein occurs in the endosperm. The effective strategy established in this study can be used in rice and possibly in other important crops.

## Results

### Vector construction, plant transformation and mechanism of the Cre-lox gene switch

**Figure 1:**
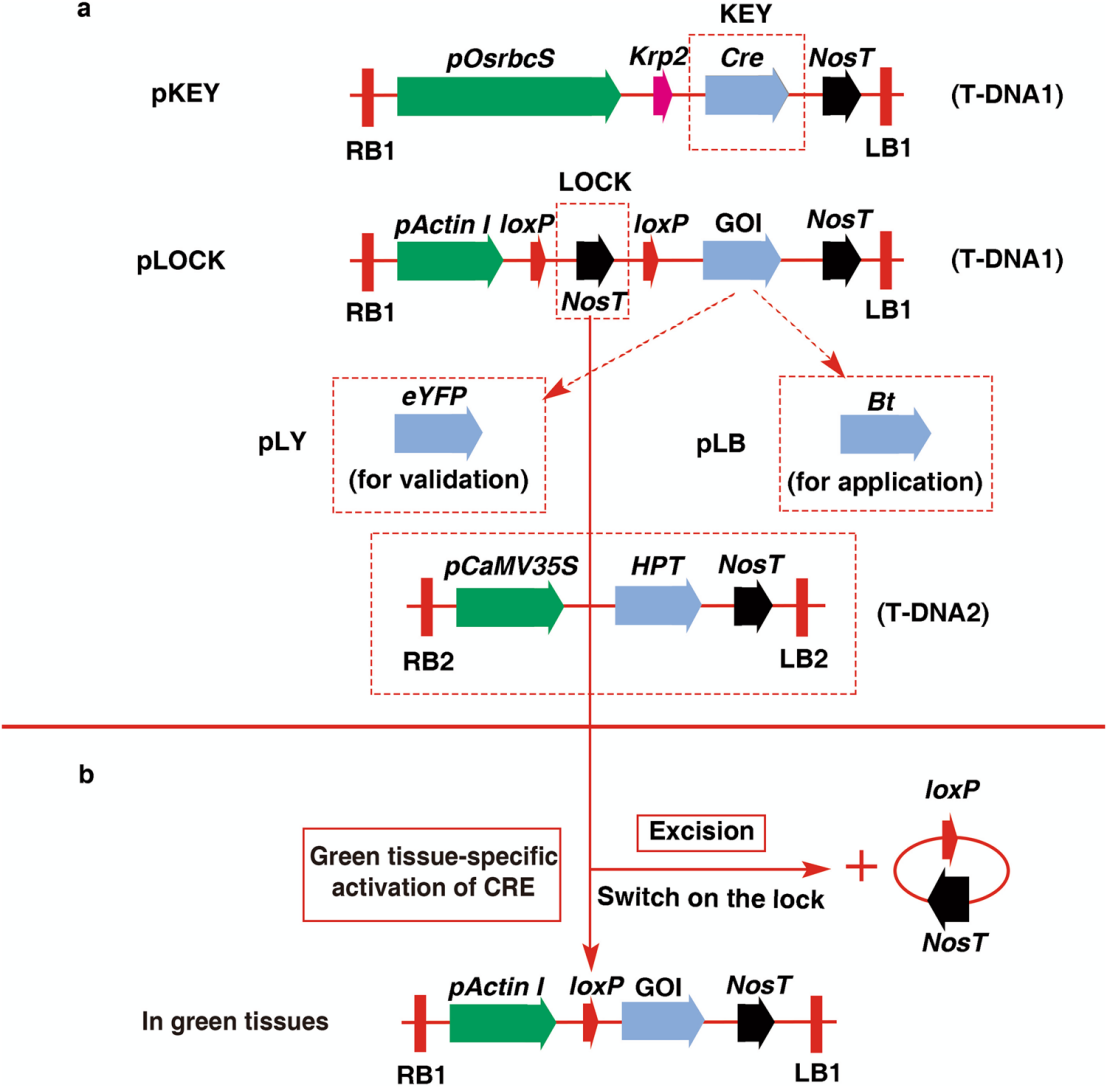
Schematic illustration of the gene cassettes and the principles of the Cre-lox gene switch technology. (**a**) Gene cassettes used in this study. The *Cre* gene fused with the NLS of *Krp2* was driven by the green-tissue-specific *rbcS* promoter in the pKEY vector; the pLOCK vector contained the rice *Actin I* promoter following a *Nos* terminator, which was bounded by two *loxP* loci as a lock to block the expression of the GOI. The eYFP and *Bt* genes were used for validation and a test of field application. These genes were subcloned into the GOI site of pLOCK, and the resulting vectors were named pLY and pLB, respectively. LB and RB, left border and right border, respectively. The *hygromycin B phosphotransferase* (*HPT*) gene in the T-DNA2 site of pSB130 functioned as a selectable marker of transformation. (**b**) Schematic illustration of the Cre-mediated excision of the genetic lock from LOCK cassette  in green tissues. Cre expression should lead to the deletion of *loxP*-*NosT*, thus switching on the normal expression of the GOI, specifically in the green tissues. However, Cre recombinase is not expressed in non-green tissues. Thus, the GOI in non-green tissues is kept locked forever, and GOI expression never occurs.

The Cre-lox gene switch requires the construction of two completely independent vectors, pKEY and pLOCK. To construct the pKEY vector, a 3179-bp segment of the *rbcS* promoter (Supplementary Fig. S1a) was obtained from the genomic DNA of wild-type Nipponbare and subcloned into multiple cloning sites (MCS) in T-DNA1 of the commercially available binary vector pSB130 (Supplementary Fig. S2), followed by a *Cre* recombinase gene tagged with the nuclear localization sequence (NLS) of *Arabidopsis Krp2* (Supplementary Fig. S1b)^36^ (Fig. 1a). Using the same method, the pLOCK vector was constructed with the rice *Actin I* promoter following a *Nos* terminator, which was bounded by two *loxP* loci as a lock to block the expression of the GOI (Fig. 1a). Here, the enhanced yellow fluorescence protein (eYFP) and *Bt* gene (*Cry1Ab/1Ac*) were used for validation and application, respectively. These genes were subcloned into the GOI site of pLOCK, and the resulted plasmids were named pLY and pLB. The *hygromycin B phosphotransferase* (*HPT*) gene in T-DNA2 of pSB130 functioned as a selectable marker for transformation (Fig. 1a) with a high chance to integrate separately from the target gene (into different chromosomes or different loci on the same chromosome) for easy removal from the offspring by selfing and segregation after transformation.

Then, the resulting constructs were introduced into the genome of wild-type Nipponbare and MH63, respectively, mediated by *Agrobacterium* EHA105^37^. The resulted transgenic lines were named KEY, LY, and LB (Nipponbare background), and M-KEY, M-LB (MH63 background) lines, respectively. Transformation of Nipponbare calli using HPT to select and resulted in a total of 41, 30 and 50 transformants of pKEY, pLY and pLB, of which 21, 12 and 22 contained the KEY, LY and LB cassette, respectively while using PCR for identification. In MH63 background, there were 10 and 30 transformants of KEY and LB cassette, of which 4 and 19 contained the KEY and LB cassette, respectively.

According to the design of the system, the GOI in the LOCK (LY, LB or M-LB) line cannot be transcribed until this line is crossed with the KEY (or M-KEY) line. At that time, the NLS-tagged Cre recombinase driven by the rice *rbcS* promoter from KEY line can excise the *loxP-*bounded *NosT* fragment from the LOCK line, which switches on the LOCK to transcribe the GOI in green tissues. The mechanism by which this *loxP*-bounded *NosT*-specific recombination mediated by Cre recombinase occurred in the green tissues of the LOCK and KEY hybrid lines is shown in Fig. 1b, and the unlocked GOI will thus be expressed as normal. However, the locked GOI in the non-green tissues is not transcribed due to the lack of Cre recombinase to open the LOCK by removing the *NosT* fragment.

### Validation of the Cre-lox gene switch via eYFP

**Figure 2:**
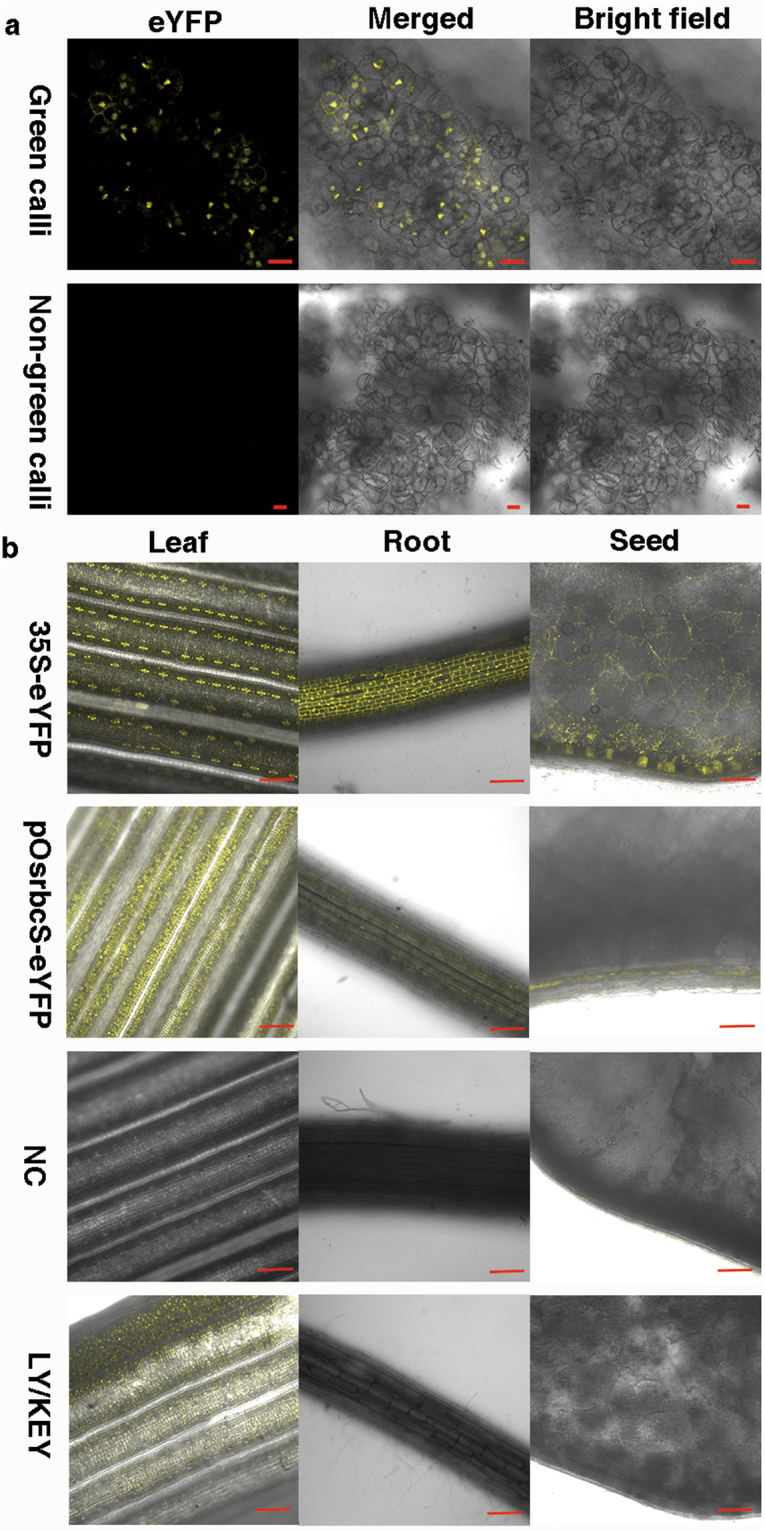
Cre-lox gene switch verification via eYFP and its tissue expression pattern. (**a**) Transformed calli positive for pLY were regenerated to green tissues on regeneration medium for thirteen days and then retransformed with pKEY for transient expression via bombardment; the Cre recombinase expression driven by the *rbcS* promoter deleted the lock in the green tissues, so the eYFP was expressed, and yellow fluorescence appeared. Transformed calli positive for pLY but consisting of non-green tissues underwent the same treatment as a negative control. Bar = 20 μm. (**b**) The tissue expression patterns of eYFP in the leaf, root, and cross section of seed in the LY/KEY hybrid lines by confocal micrography. 35S-YFP and pOsrbcS-eYFP lines were used as positive controls, while wild-type Nipponbare and the KEY and LY lines were used as negative controls (NC). Bar = 100 μm.

To verify the function of the Cre-lox gene switch, the pKEY plasmid was introduced into pLY-positive calli via biolistic transformation after or without regeneration. The calli after regeneration contained green tissues. Twenty-four hours after transformation, detectable eYFP signal had appeared in the green post-regeneration calli; in contrast, no signals were detected in the non-regenerated calli, which lacked green tissue (Fig. 2a). These results preliminarily confirmed that the Cre-lox gene switch works in green tissues only.

We further observed eYFP signals under the control of the Cre-lox gene switch in leaf and root tissues and in cross sections of three-day germinating seeds collected from the F3 progeny, which were confirmed to be homozygous for both LOCK and KEY genes, of 6 LY/KEY (LYi/KEYj; i = 3, 4, 5; j = 1, 2) hybrids. The typical signal patterns are shown in Fig. 2b. The positive control lines harbouring *35 S*::*eYFP* showed constitutive fluorescence in all tissues, while the positive control lines with pOsrbcS-eYFP exhibited a different pattern of fluorescence, i.e., extensive signal appeared in the green tissues but only faint signals were present in root tissues and the aleurone cells of seeds. The LY/KEY F3 progeny showed eYFP signals similar to those of both positive controls in the leaf tissues, but a complete absence of signal in either roots or seeds, similar to the negative controls (Fig. 2b) (wild-type control, the KEY and LY lines), which contained no eYFP protein (Supplementary Fig. S1c).

**Figure 3:**
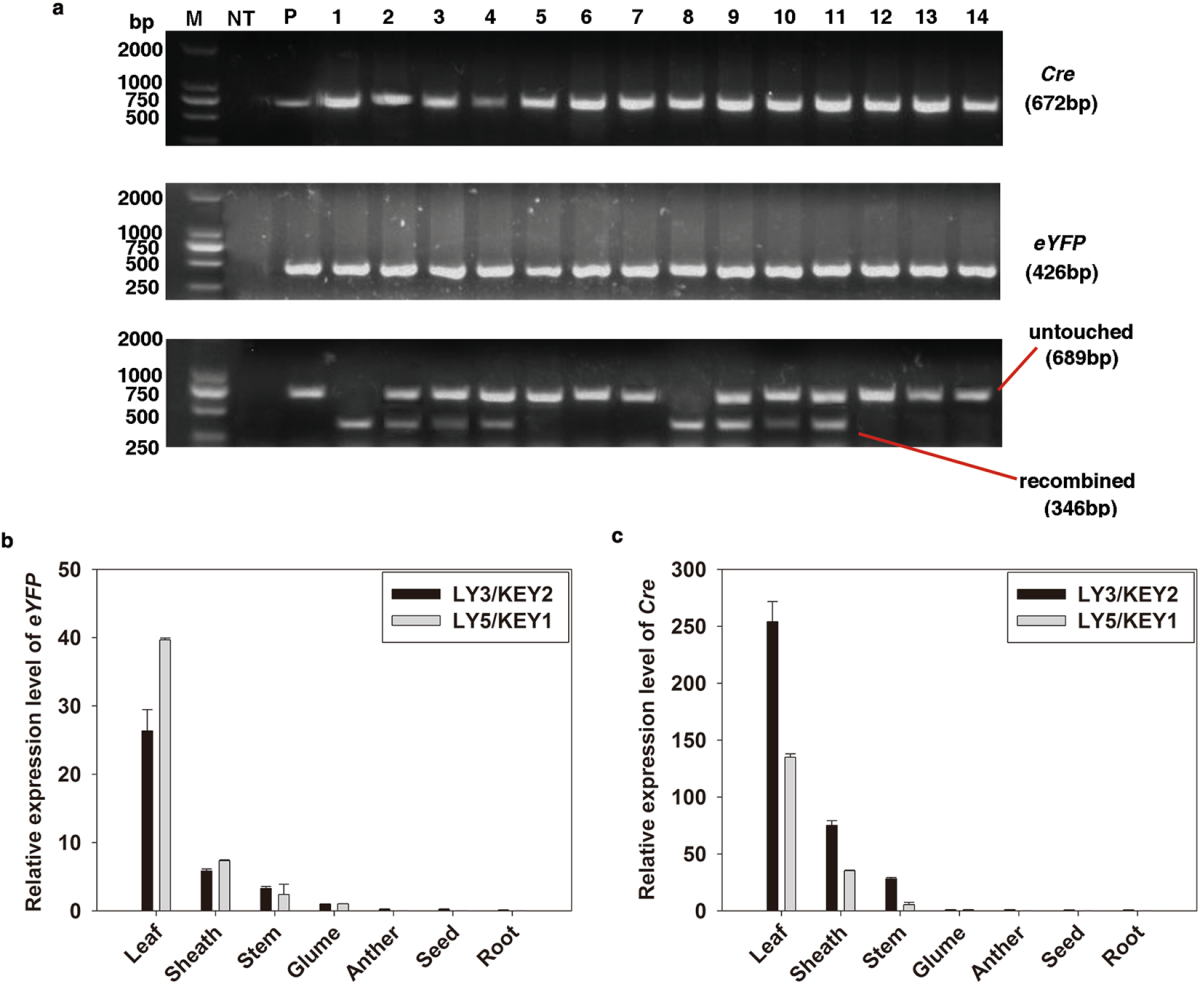
Detection of Cre recombinase-mediated recombination events and expression pattern analyses of eYFP and Cre in LY/KEY hybrid lines. (**a**) *eYFP* and *Cre* DNA were present in all green and non-green tissues of the LY/KEY hybrid lines, but the DNA recombination mediated by Cre recombinase was detected in the green tissues only. P indicates pLY, which served as a control in which the genetic lock was untouched in the absence of Cre recombinase; NT indicates non-transgenic plants; and 1–7 and 8–14 indicate the leaf, sheath, stem, glume, anther, seed and root of the LY3/KEY2 and LY5/KEY1 lines, respectively. (**b**) and (**c**) The relative expression levels of eYFP and Cre in the LY3/KEY2 and LY5/KEY1 lines, respectively, as detected by qRT-PCR. Error bars indicate the SD based on three replications.

To demonstrate the presence of the KEY and LY genes without site-specific recombination in the tissues that did not display eYFP signals, we performed PCR analysis using two F3 progeny derived from the LY3/KEY2 and LY5/KEY1 hybrids. The expected bands representing KEY and LY not only appeared in the green tissues of the leaf, sheath, stem and glume but also in the non-green anther, root and seed organs, and site-specific recombination had occurred in all detected green tissues and in those tissues only (Fig. 3a). However, the frequency of recombination varied with the content of chlorophyll in different tissues. For example, the frequency of recombination in the leaf tissue, which has the highest chlorophyll content, reached almost 100%, while that in the sheath, stem and glume tissues, which have lower chlorophyll content, was approximately 50% or slightly lower than 50%. However, the frequency of recombination in the anther, seed and root organs that contain no chlorophyll was zero (Fig. 3a). These results were consistent with data obtained by qRT-PCR analysis, which showed expression of both eYFP and Cre in green tissues only (Fig. 3b).

### Validation of the Cre-lox gene switch via application of the *Bt* gene

**Figure 4:**
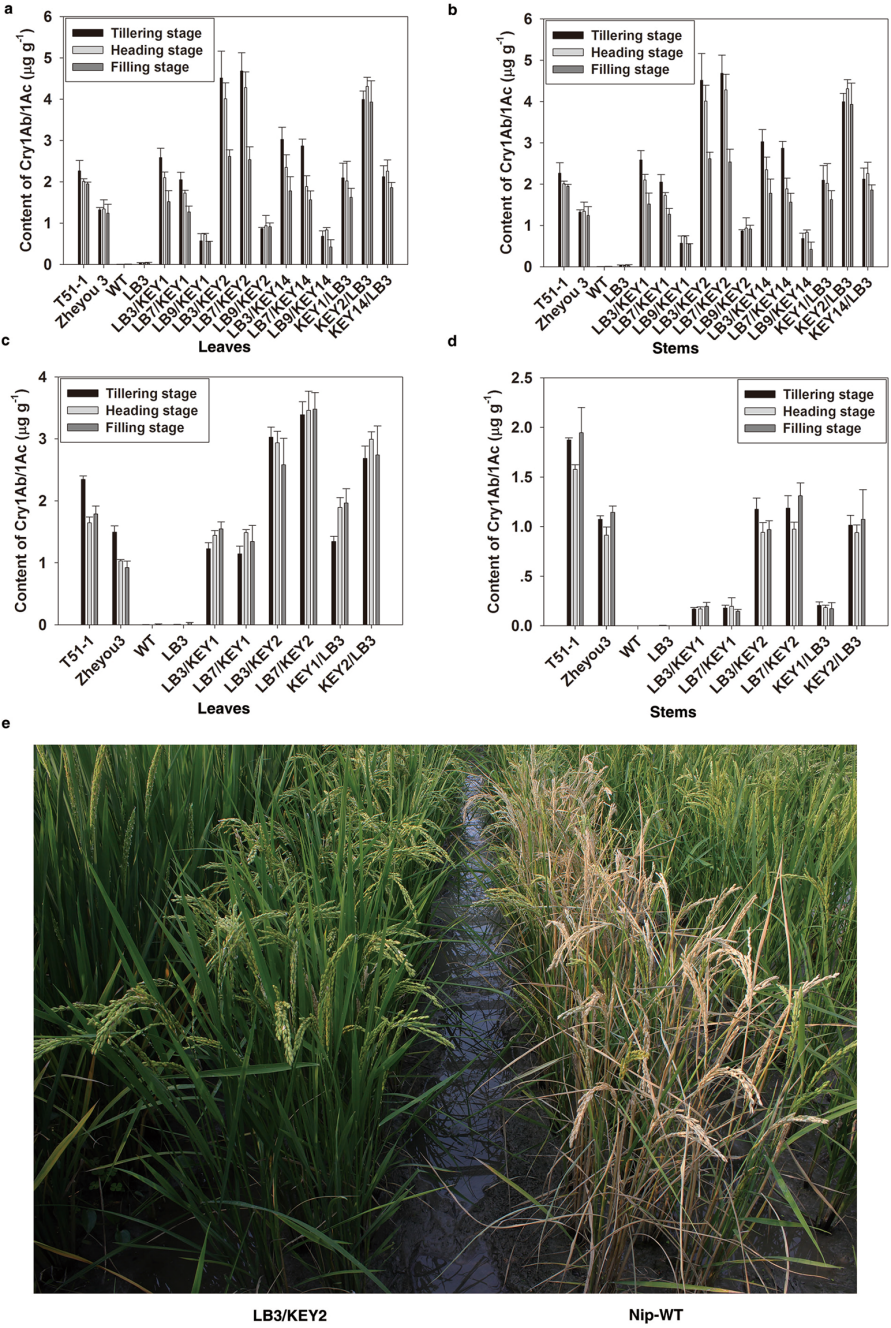
The contents of Cry1Ab/1Ac protein in the LB/KEY and KEY/LB lines and the field performance of the LB3/KEY2 line against a manual infestation of striped stem borers. (**a**,**b**,**c** and **d**) The contents of Cry1Ab/1Ac protein in the leaves and stems of the LB/KEY and KEY/LB lines in 2014 and 2015, respectively. Error bars indicate SD based on three biological replications and two technological replications. T51–1 and Zheyou 3 were used as positive controls, and the parental LB line and wild-type Nipponbare were used as negative controls. (**e**) The field performance of LB3/KEY2 against a manual infestation of striped stem borers. Wild-type Nipponbare was used as a negative control.

**Figure 5:**
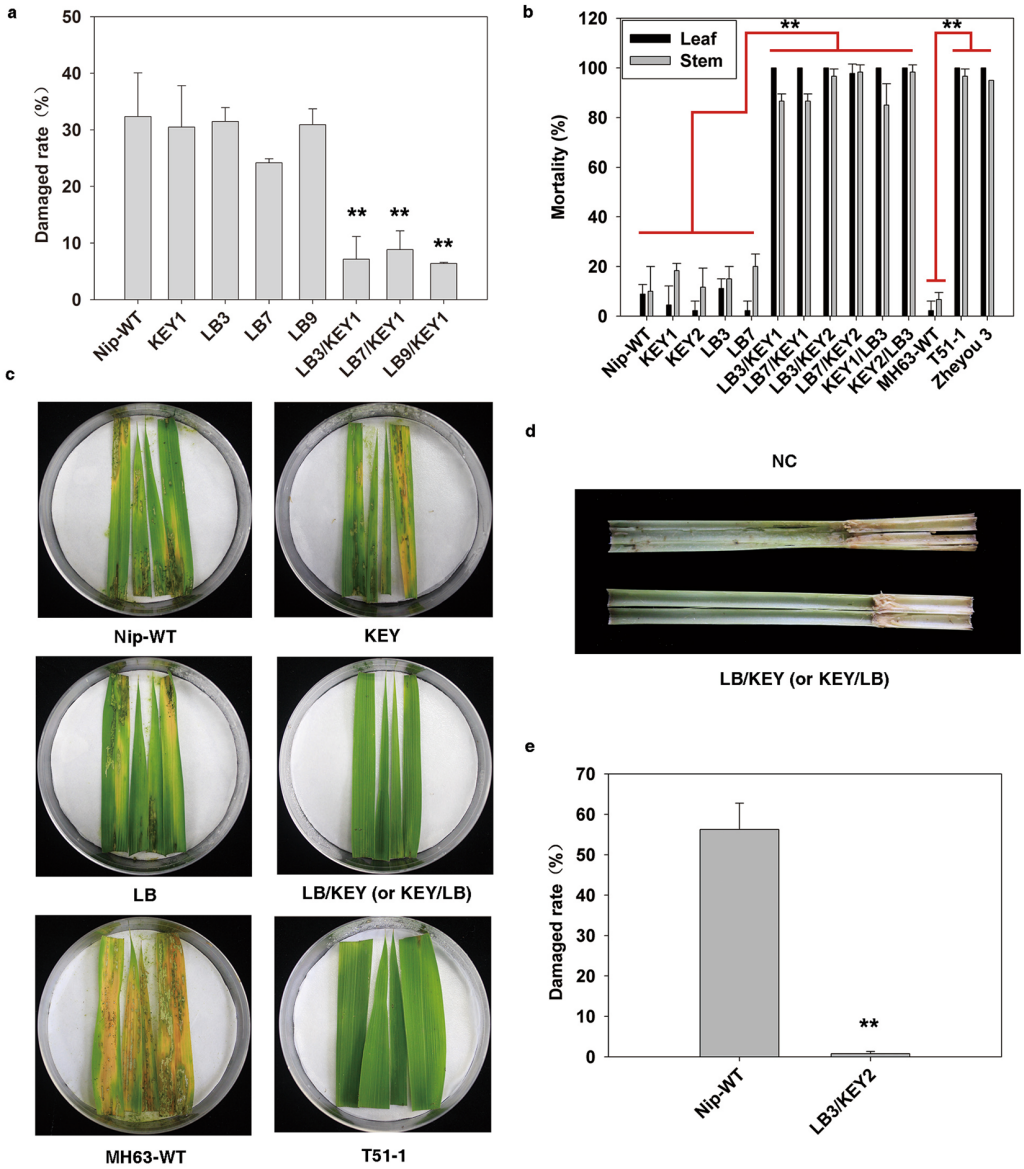
Resistance of the LB/KEY and KEY/LB hybrid lines to manual infestations of striped stem borers. (**a**) Insect resistance evaluation of the hybrids LB3/KEY1, LB7/KEY1 and LB9/KEY1 (with low Cry1Ab/1Ac protein content) carried out under field conditions in 2014. Each line was tested for three replications, and each replication contained 10 individual plants. (**b**) The mortality of larvae fed on leaf and stem sections from different tested lines during a manual infestation of striped stem borers under laboratory conditions; each line was tested in three replications. (**c**) Typical resistance of hybrid leaf sections to a manual infestation of striped stem borers under laboratory conditions. The leaf sections of T51-1 were used as a positive control. The leaf sections of the parental lines KEY and LB and the wild types Nipponbare and MH63 were used as negative controls. (**d**) Typical resistance of stem sections from the hybrid LB/KEY lines and the negative controls against a manual infestation of striped stem borers under laboratory conditions. (**e**) Insect resistance evaluation of the LB3/KEY2 hybrid (with a higher Cry1Ab/1Ac protein content) carried out in 2015. Wild-type Nipponbare was used as a negative control. Each line was tested for three replications, and each replication contained 24 individual plants. P values were generated by the Dunnett t test, and **P < 0.01 denotes statistical significance.

To further verify whether the Cre-lox gene switch can specifically and efficiently express the transgenic trait in rice green tissues, 3 LB lines containing single copies of LB cassette were crossed with 3 KEY lines containing single copies of KEY cassette  (verified by *Southern* blot) (Supplementary Fig. S3a,b) to make 12 LB/KEY and KEY/LB lines (LBm/KEYn, KEYn/LBm; m = 3, 7, 9; n = 1, 2, 14). Among them, KEY1, KEY2, LB3 and LB7 were antibiotic marker (*HPT*)-free lines (Supplementary Fig. S3c). Before the field evaluation of insect resistance in 2014, Cry1Ab/1Ac protein contents were determined in the leaves and stems by enzyme-linked immunosorbent assay (ELISA) as shown in Fig. 4a,b. The results showed that most of the LB/KEY and KEY/LB lines contained the Cry1Ab/1Ac protein in their leaves at levels as high as those of the positive controls (T51-1 and its derivative hybrid Zheyou 3^38^) in all three growth stages (tillering, heading and filling). However, the Cry1Ab/1Ac protein content of these hybrids was lower than that of the two positive controls in the stems, except for KEY2/LB3, KEY2/LB7, and LB3/KEY2, which had similar contents to that of Zheyou 3^38^. Based on these results, the LB3/KEY1, LB7/KEY1 and LB9/KEY1 lines with low Cry1Ab/1Ac protein contents due to low *Cre* expression levels (Supplementary Fig. S4a) were further selected to determine whether they had good resistance against a manual infestation of striped stem borers. The KEY and LB parental lines and wild-type Nipponbare, which contained no Bt protein (Supplementary Fig. S4b), were used as negative controls. The results indicated that the negative controls were severely damaged by striped stem borers, and the number of damaged tillers per plant varied from 23.92% to 30.65%. The LB3/KEY1, LB7/KEY1 and LB9/KEY1 lines experienced only 6.40% to 7.64% damage, which was significantly (P < 0.01) lower than that of the negative control lines (Fig. 5a). Typical phenotypes are shown in Supplementary Fig. S4c. The insect resistance of the other 9 LB/KEY and KEY/LB lines were also evaluated against natural outbreaks of striped stem borers. Severe damage was observed in the negative controls, with a rate between 6.96% and 9.85%, while the hybrid LB3/KEY2, LB7/KEY2, and KEY2/LB3 lines showed no damage from the striped stem borers, and the worst damage rate in the KEY1/LB3 line was only 0.63% (Table 1). These data thus imply that LB/KEY and KEY/LB hybrid lines under the control of the Cre-lox gene switch, even with a minimal content of Cry1Ab/1Ac protein, had a much higher resistance against striped stem borers than the negative controls.

**Table 1:** Resistance reaction of the parental KEY and LB lines, the hybrid LB/KEY and KEY/LB lines and the wild-type Nipponbare against striped stem borers and leaffolders under field conditions (Hangzhou, China).

Lines	2014			2015		
	Damaged by striped stem borers (%)	Damaged by leaffolders (%)		Damaged by striped stem borers (%)	Damaged by leaffolders (%)	
		The rate of tillers damaged per plant (%)	Number of leaves damaged per plant		The rate of tillers damaged per plant (%)	Number of leaves damaged per plant
Nip-WT	7.59 ± 0.69	63.82 ± 10.70	12.47 ± 1.20	7.22 ± 1.00	59.76 ± 6.14	12.17 ± 0.57
KEY1	ND	49.64 ± 2.85*	9.83 ± 0.61	7.63 ± 1.07	60.11 ± 0.73	11.07 ± 0.15**
KEY2	6.96 ± 0.79	54.81 ± 0.93	9.17 ± 0.91**	7.46 ± 1.39	56.71 ± 7.69	10.70 ± 0.61**
KEY14	9.85 ± 1.98**	59.52 ± 6.53	9.80 ± 1.74**	ND	ND	ND
LB3	8.02 ± 0.77	65.27 ± 8.19	12.47 ± 1.20	6.73 ± 0.56	62.33 ± 2.09	11.67 ± 0.42
LB7	7.39 ± 0.47	58.36 ± 3.59	14.60 ± 0.62**	7.02 ± 0.41	61.49 ± 1.29	13.37 ± 0.67**
LB9	9.12 ± 0.39*	64.18 ± 1.27	10.83 ± 0.31**	ND	ND	ND
LB3/KEY1	ND	0.65 ± 1.12**	0.13 ± 0.23**	0.28 ± 0.48**	0.00 ± 0.00**	0.00 ± 0.00**
LB7/KEY1	ND	1.33 ± 2.31**	0.13 ± 0.23**	0.00 ± 0.00**	0.25 ± 0.44**	0.03 ± 0.06**
LB9/KEY1	ND	0.00 ± 0.00**	0.00 ± 0.00**	ND	ND	ND
LB3/KEY2	0.00 ± 0.00**	0.31 ± 0.53**	0.03 ± 0.06**	0.00 ± 0.00**	0.26 ± 0.45**	0.00 ± 0.00**
LB7/KEY2	0.00 ± 0.00**	0.00 ± 0.00**	0.00 ± 0.00**	0.00 ± 0.00**	0.00 ± 0.00**	0.00 ± 0.00**
LB9/KEY2	0.62 ± 0.54**	0.00 ± 0.00**	0.00 ± 0.00**	ND	ND	ND
LB3/KEY14	1.63 ± 0.66**	0.91 ± 1.57**	0.07 ± 0.12**	ND	ND	ND
LB7/KEY14	1.16 ± 1.33**	0.29 ± 0.50**	0.03 ± 0.06**	ND	ND	ND
LB9/KEY14	1.00 ± 1.02**	0.32 ± 0.56**	0.03 ± 0.06**	ND	ND	ND
KEY1/LB3	0.63 ± 0.55**	0.63 ± 0.55**	0.07 ± 0.06**	0.00 ± 0.00**	0.26 ± 0.45**	0.07 ± 0.12**
KEY2/LB3	0.00 ± 0.00**	0.00 ± 0.00**	0.00 ± 0.00**	0.00 ± 0.00**	0.00 ± 0.00**	0.00 ± 0.00**
KEY14/LB3	1.54 ± 0.59**	1.22 ± 1.37**	0.10 ± 0.10**	ND	ND	ND

All dada were measured from 10 randomly sampled plants per test material per replication (3 replications) and were analysed by the Dunnett t test using the SPSS 22.0 software package. Values are given as the mean ± standard deviation (SD). Nip-WT (wild-type Nipponbare) as the negative control; all of the others lines were compared with the Nip-WT. ND means not detected. * and ** meant significantly different from the control at P < 0.05 and P < 0.01, respectively.

When subjected to a natural infestation of leaffolders, all 12 hybrid lines showed significantly reduced damage compared with that of the negative controls. The rate of tillers damaged and the number of leaves damaged per hybrid plant were 0 to 1.33% and 0–0.13, while those of the negative controls were 49.64–65.27% and 9.17–14.60, respectively (Table 1). These data thus demonstrate that the Cre-lox gene-switch-controlled *Bt* rice hybrids were highly resistant to leaffolders under field conditions.

To further address whether the Cre-lox gene switch can strictly prevent the expression of the transgene in seeds, the Bt protein from endosperm and brown rice was quantified using ELISA. As shown in Table 2, the detected value of Cry1Ab/1Ac protein in the endosperm showed no differences between all hybrid lines and wild-type Nipponbare, while that in the brown rice collected from the lines LB3, LB3/KEY2, LB7/KEY2 and KEY2/LB3 was only slightly higher than that of wild-type Nipponbare, possibly due to the effect of the rice seed coat, the embryo or both. However, the positive controls T51-1 and Zheyou 3 had, respectively, 371.67 ng g^−1^ and 157.32 ng g^−1^ Cry1Ab/1Ac proteins in endosperm and 897.36 ng g^−1^ and 400.53 ng g^−1^ Cry1Ab/1Ac proteins in brown rice. These results thereby confirmed that the Cre-lox gene switch could indeed completely switch off the expression of the GOI in rice endosperm.

**Table 2:** Cry1Ab/1Ac protein assay of the endosperm and brown rice of the parental LB line, the hybrid LB/KEY and KEY/LB lines, the positive control T51-1 and Zheyou3 lines and negative control wild-type Nipponbare quantified by ELISA.

Lines	the content of Cry1Ab protein (ng g^−1^)			
	2014		2015	
	In endosperm	In brown rice	In endosperm	In brown rice
Nip-WT	0.42 ± 0.08	0.71 ± 0.11	0.21 ± 0.07	0.24 ± 0.11
LB3	0.67 ± 0.37	1.36 ± 0.59**	0.22 ± 0.08	1.81 ± 0.48**
LB3/KEY1	0.44 ± 0.06	1.07 ± 0.13	0.23 ± 0.08	0.33 ± 0.03
LB7/KEY1	0.44 ± 0.04	0.86 ± 0.09	0.17 ± 0.05	0.61 ± 0.25
LB9/KEY1	0.38 ± 0.06	0.85 ± 0.06	ND	ND
LB3/KEY2	0.80 ± 0.20	2.09 ± 0.15**	0.25 ± 0.02	1.19 ± 0.49*
LB7/KEY2	0.64 ± 0.10	2.52 ± 0.06**	0.28 ± 0.12	1.06 ± 0.27
LB9/KEY2	0.51 ± 0.12	1.58 ± 0.13**	ND	ND
LB3/KEY14	0.51 ± 0.09	1.18 ± 0.18*	ND	ND
LB7/KEY14	0.46 ± 0.11	1.40 ± 0.09**	ND	ND
LB9/KEY14	0.41 ± 0.08	1.27 ± 0.23**	ND	ND
KEY1/LB3	0.29 ± 0.05	1.04 ± 0.01	0.14 ± 0.08	0.20 ± 0.05
KEY2/LB3	0.60 ± 0.13	1.98 ± 0.17**	0.25 ± 0.08	1.50 ± 0.58**
KEY14/LB3	0.35 ± 0.09	1.06 ± 0.07	ND	ND
T51-1	371.67 ± 30.85	879.36 ± 67.71	305.23 ± 27.44	1040.33 ± 60.38
Zheyou 3	157.32 ± 27.2	400.53 ± 34.79	212.17 ± 47.27	578.69 ± 1.10

All data were measured from 50 randomly sampled seeds per test material per replication (3 replications) and analysed by the Dunnett t test using the SPSS 22.0 software package. The values were given as the mean ± SD. Nip-WT as the negative control; all of the other lines were compared with it. ND meant not detected. * and ** meant significantly different from the control at P < 0.05and P < 0.01, respectively.

The agronomic performance data collected from the field experiment in 2014 showed that the LB3/KEY2, LB7/KEY2, LB7/KEY14, and KEY2/LB3 lines were not significantly different from wild-type Nipponbare, and the LB3/KEY2 line had slightly more grains per panicle than the control. The other LB/KEY and KEY/LB lines had various degrees of differences in agronomic traits, especially plant height (Table S1). These results showed that LB3/KEY2, LB7/KEY2, and KEY2/LB3 are the ideal lines for Cre-lox gene switch-controlled Bt application, as they had good resistance against lepidopteran pests, with no Bt protein in seeds, and had good agronomic traits and yield performance in the field.

### Repeated verification of the Cre-lox gene switch via application of the Bt gene

To further confirm the above results, the above 3 ideal lines and the LB3/KEY1, LB7/KEY1 and KEY1/LB3 lines were selected to perform the same experiments in 2015 to further verify the Cre-lox gene switch. Similar to the results obtained in 2014, the 6 tested hybrids stably expressed the Cry1Ab/1Ac protein. All had high protein content in their leaves and stems compared with the positive control (Fig. 4c and d) and an undetectable amount in their endosperm with no difference from wild-type Nipponbare (Table 2). The resistances of these hybrids to the natural outbreaks of both striped stem borers and leaffolders were also much better than those of the negative controls (Table 1). Additionally, all these 6 hybrids were selected for laboratory tests against a manual infestation of striped stem borers; the mortality of larvae feeding on the leaf and stem sections increased from 2.22 to 11.11% in the leaves of the negative controls and from 10.00–20.00% in the stems of the negative controls to more than 97.78–100.00% and 85.00–98.33% in the leaves and stems of the hybrids, respectively (Fig. 5b). Typical phenotypes are shown in Fig. 5c and d. Specifically, the hybrid LB3/KEY2, which had a higher Cry1Ab/1Ac protein content in its leaves and stems in both years, was selected for field tests against a manual infestation of striped stem borers. Wild-type Nipponbare suffered serious damage caused by this manual infestation under field conditions compared to LB3/KEY2 (Fig. 4e); the damage rate decreased from 56.26% in the wild type to 0.78% in the LB3/KEY2 plants (Fig. 5e). Therefore, these results confirmed a second time that the Cre-lox gene switch could efficiently express the GOI in rice green tissues but not produce any GM protein in its endosperm. Furthermore, the agronomic performance of LB3/KEY2 and its parental lines KEY2 and LB3 were not significantly different from that of wild-type Nipponbare in the field test in Hangzhou (Table S2).

### Cre-lox gene switch also functions in the *indica* rice background

To test whether the Cre-lox gene switch was functional in the *indica* rice cultivar, two hybrids, M-LB10/M-KEY4 and M-LB10/M-KEY6, were used for experiments in 2015. Similar to the results observed in the *japonica* hybrids, the two tested *indica* hybrids stably expressed the Cry1Ab/1Ac protein in leaves and stems and had an undetectable amount in seeds with no difference from the negative control (Supplementary Fig. S5). Their resistance against the natural outbreaks of both striped stem borers and leaffolders were also much better than that of the wild-type control and parental lines (Table S3). Larvae feeding on the leaf and stem sections of the M-LB/M-KEY lines showed significantly higher mortality than those feeding on the negative controls (Supplementary Fig. S6a,b and c). Meanwhile, the selected M-LB10/M-KEY6 line, which had higher Cry1Ab/1Ac protein content in its leaf and stem tissues, also displayed excellent resistance against a manual infestation of striped stem borers under field conditions. The damage rate was reduced from 81.36% in the wild-type control to 4.40% in M-LB10/M-KEY6 (Supplementary Fig. S6d and e). Finally, no negative alteration of agronomic traits was observed in the M-LB10/M-KEY6 hybrids (Table S4). Therefore, we can conclude that the Cre-lox gene switch works well in both *japonica* and *indica* rice cultivars.

## Discussion

Striped stem borers and leaffolders, which are two lepidopteran pests of rice, cause severe yield losses in most rice-producing countries^7,38^. The Cre-lox gene-switch-controlled *Bt* rice, including 12 LB/KEY and KEY/LB hybrids, that we developed in this study showed good resistance to these two destructive pests. The Bt content detected in the leaves of these 12 hybrids was as high as 0.42–4.68 µg/g, which is comparable to that of the green-tissue-specific promoter (*rbcS*)-driven Bt rice developed previously^7,39^. Compared to those in the leaves, the detected Bt contents in the stems were not high in all LB/KEY or KEY/LB hybrids, but the Bt contents in the stems of some lines, such as LB3/KEY2, LB7/KEY2 and KEY2/LB3, were comparable to that in the stems of Zheyou 3^38^, a T51–1 derivative line carrying an *Actin I* promoter-driven *Bt* gene.

As for the Bt contents in the endosperm, the previously reported tissue-specific promoters could reduce them to very low levels^7,11,14,39–41^ but not to zero, due to background expression of the promoters. For instance, the famous Bt rice RJ5, which harbours an *rbcS* promoter-driven *Cry1C* construct, can only reduce endosperm Bt protein to 2.6 ng g-1^7^. In contrast, the improved Cre-lox gene switch in this study further reduced the Bt content in the endosperm by an order of magnitude, that is, to the same level as in the wild-type control (Table 2). These results thus imply that the Cre-lox gene switch is an effective tool not only for expressing transgenic traits in rice green tissues but also for preventing the production of GM protein in the endosperm.

**Figure 6:**
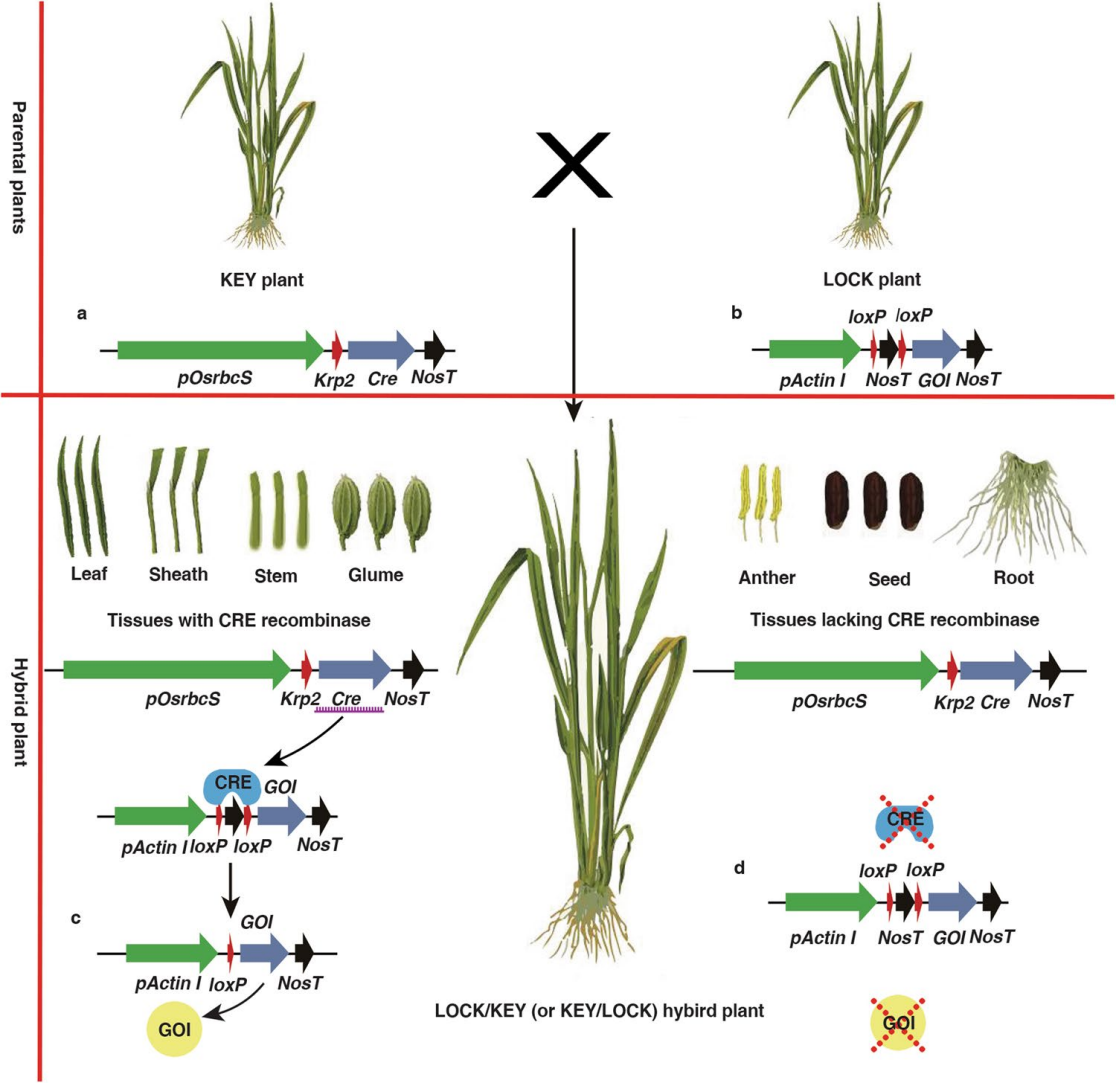
Schematic illustration of the production of plants free from GM proteins in non-green tissues from two GM parental plants using the Cre-lox gene switch. The upper panel shows the use of the Cre-lox gene switch system to produce KEY and LOCK parental plants. The lower panel shows that if these two plants undergo pyramiding to obtain LOCK/KEY or KEY/LOCK hybrids, the *NosT*-*loxP* DNA sequence representing the genetic lock will be deleted from tissues in which the recombinase is expressed. When a green-tissue-specific gene promoter is used to limit recombinase expression to green tissues, such as the leaf, sheath, stem, and glume, the genetic lock should be deleted from these tissues, resulting in normal expression of the GOI. However, CRE recombinase is not expressed in the root, anther or seed; in these tissues, the GOI is kept locked, and its function remains untouched forever. Thus, the Cre-lox gene switch system should provide a useful tool to develop GM crop plants that are free of GM protein in the edible parts, and this technique may also have many other applications, as described in the ‘Discussion’ section. (**a**) The T-DNA region of the integrated construct pKEY in the KEY line. (**b**) The T-DNA region of the integrated construct pLOCK in the LOCK line. (**c**) The T-DNA region of the integrated construct pLOCK was switched on in the green tissues of the LOCK/KEY or KEY/LOCK hybrid lines, where Cre recombinase is active. (**d**) The T-DNA region of the integrated construct pLOCK in the non-green tissues of the hybrid line was unchanged, due to the absence of Cre recombinase.

The reasons that the Cre-lox gene switch can control tissue-specific expression of the GOI so thoroughly are as follows: First, in the Cre-lox gene switch, the expression of Cre recombinase is designed to be driven by the green-tissue-specific promoter *rbcS*. This restriction means that the Cre recombinase is highly expressed in green tissues but is expressed only at a background level in the non-green tissues, including the endosperm. Second, the fusion of an *Arabidopsis* nuclear localization signal peptide derived from the *Krp2* gene to Cre recombinase causes the efficient import of this recombinase into the nucleus, where it induces site-specific recombination, and thus may enhance the efficiency of recombination. Third, the recombination rate of Cre recombinase drops markedly in the non-green tissues due to the lack of excess enzyme molecules acting on the *loxP* loci. This situation is even more pronounced in the endosperm, since the small amount of Cre recombinase expressed there is likely degraded along with the programmed degradation of the endosperm cell nucleus during the development of the endosperm^42–46^, which thus further reduces the rate of Cre-mediated recombination to zero. A flowchart explaining the mechanism and effects of the Cre-lox gene switch is shown in Fig. 6.

In addition to the Cre-lox gene switch that we constructed in this study for preventing transgene expression in the endosperm of the seed plant, several similar technologies for transgene containment with pollen or seeds as target have also been developed previously. These technologies include “GM-gene-deletor”^47^, which was developed to eliminate all transgenes in target organs or entire plants; “terminator seed technology”^48^, which was used to prevent the seed-mediated spread of GM genes; and “male and female sterility technology”^49^, a technology for reducing the problem of gene flow from vegetatively propagated trees. Compared with these traditional techniques, our Cre-lox gene switch is technically unique. First, the technology’s goal is to completely shut down the expression of the GM gene in seeds, without removal of the DNA. Therefore, this technique does not share the problem of the “GM-gene-deletor” technology, that is, once the transgene is deleted, it no longer exists, so that the “GM-gene-deletor” technology can be only applied to asexually propagated crops^47^. Theoretically, the Cre-lox gene switch has no limits to its application either in sexually or asexually reproducing crops. Second, the design strategy used to realize the technical goal in the Cre-lox gene switch was to first turn off all gene expression and then to activate expression in only the desired locations. Simply because of this, there is no “leakage problem”, which tissue-specific promoters inevitably have due to background expression^7^. Third, our Cre-lox gene switch is composed of two independent constructs, which can be transformed either into different recipient genomes, such as two parental lines, and then integrated into F_1_ hybrids through sexual hybridization, or into the same genetic background in different plants, which can then be combined into a single line to create conventional varieties through backcrossing and marker-assisted selection.

As mentioned above, the antibiotic marker gene used for transformant screening in this study was constructed using a different vector T-DNA region from that of the target gene; therefore, the marker has a high chance to integrate independently from the desired transgene, into different chromosomes or different loci of the same chromosome, making it easy to remove through selfing segregation of offspring^50^. In fact, according to our design, all antibiotic marker genes in any transgenic material that has a good prospect of application must be removed, so that no hidden threats to biological safety exist in this regard.

As far as the application of the Cre-lox gene switch is concerned, some hybrids or their offspring bred in this study have shown good development potential. One example is the LB3/KEY2 hybrid line; not only do its stems and leaves exhibit excellent insect resistance equally as effective as that of traditional Bt rice and its seeds not accumulate any GM protein but also its main agronomic traits are not different from those of the wild-type control (Table S1 and Table S2). These results indicate that the Cre-lox gene switch has no negative impacts on the agronomic performance of its breeding line. In summary, because no GM protein accumulates in the seeds, the implementation of Cre-lox gene switch technology could relieve public concerns about the biosafety of GM rice.

## Materials and Methods

### Plant materials and gene used

All of the transgenic plants were based on either the *Oryza sativa* ssp. *japonica* cv. Nipponbare or *Oryza sativa* ssp. *indica* cv. MH63 background. Gene Accession numbers: *pOsrbcS* (AP014968.1); *Krp2* (NM_114923.4); *Cre* (AB449974.1); *Cry1Ab/1Ac* (EU880444.1); *eYFP* (AY189981.1).

### PCR and Southern blot analyses

The PCR and *Southern blot* analyses were used to probe for the presence of the KEY, LY, and LB transformation insertion into the genome and the copy number in transgenic plants. PCR analyses were performed using the primers *Cre*-F&R, *eYFP*-F&R (*Bt*-F&R) and *HPT*-F&R (Table S5) to identify KEY, LY, and LB with *HPT*-free lines, respectively, PCR analyses were as well as utilized for validating the DNA recombination in LY/KEY hybrid lines by using the primers *eYFP*-switch-F&R (Table S5). A 20 µL mixture of 30–200 ng of template DNA, 1 × Buffer (50 mM KCl, 10 mM Tris-HCl, 0.1% Triton X-100, 2 mM MgCl_2_), 0.15 mM dNTPs, 0.1 µM each primer and 1 U of rTaq DNA polymerase (Takara, Japan) was prepared for the PCR assays. The PCR conditions were 95 °C for 3 min; 30 cycles of 95 °C for 30 min, 55 °C for 30 sec, and 72 °C for 1 min; and 72 °C for 7 min. The PCR products were then checked by electrophoresis. The plant genomic DNA was extracted by the CTAB method. For Southern blot analysis, 10 µg of genomic DNA from each sample was digested with *Hin*d III, separated on a 1% agarose gel and then transferred to a nylon membrane (GE Healthcare, UK). The probes were prepared from a PCR-amplified fragment of *Cre* and *Bt* using the DIG DNA Labelling Mix (Roche, Mannhem, Germany). All of the procedures for hybridization were performed according to the manual.

### Selection of homozygous KEY and LB lines with a single-copy insertion and obtaining the LY/KEY, LB/KEY and KEY/LB hybrid lines

The T_2_ generation of the KEY, LY, and LB transformants (KEY and LB transformants with a single-copy insertion, identified by Southern blot) was grown on the experimental farm of Zhejiang University in Hangzhou, China. Twenty-four individual plants in each T_2_ family line were used for PCR to select homozygotes containing the *Cre*, or *eYFP*, or *Bt* gene. If all 24 plants contained the target gene, we considered this line to be homozygous. The obtained LY and LB homozygotes were used as the female parent and were emasculated with hot water to cross with KEY homozygotes to generate the LY/KEY and LB/KEY lines. In contrast, the obtained KEY homozygote was used as the female parent to cross with the LB homozygote to generate the KEY/LB lines.

### Tissue localization of the Cre-lox gene switch controlled eYFP in the LY/KEY line

The plants of the LY/KEY line were used for tissue localization of the Cre-lox gene-switch-controlled eYFP. Seedlings were grown in Yoshida solution^51^ for approximately 2 weeks in a light room (temperature 25 °C; humidity 40%; light ≥3000 lux; 12 hours of light and 12 hours of night) with uniform wind circulation. The leaves and roots of the young seedlings at the 2–3 leaf stage and the cross section of the newly germinated seeds derived from the LY/KEY hybrid (after two generations of selfing to get F_3_ progeny confirmed homozygous for both the KEY and LY transgenes) were used as the objects of this study. The corresponding samples from 35S-eYFP and pOsrbcS-eYFP plants were used for comparison. The fluorescence signals were visualized under confocal microscopy (Zeiss LSM710).

### RNA extraction and qRT-PCR analysis

Total RNA was isolated from fresh plant tissues using TRIzol reagent (Invitrogen, USA). Each sample consisted of mixed RNA extracted from more than 20 plants. First-strand cDNA was generated using the Perfect Real Time Primescript RT reagent (TaKaRa, Japan) according to the kit manual. Real-time quantitative PCR was performed on an optical 96-well plate with a LightCycler 96 Real-Time PCR System (Roche, Switzerland) using SYBR Premix Ex Taq (TaKaRa, Japan) with three technical replicates. The PCR thermal cycling protocol was as follows: 95 °C for 10 s, followed by 40 cycles at 95 °C for 5 s and 60 °C for 30 s. *Actin* gene was used as the internal reference to assay the relative expression levels of *Cre* and *eYFP*. The gene-specific primers used for qRT-PCR are listed in Table S5.

### Quantification assays of the insecticidal Cry1Ab/1Ac protein

A modified procedure using the ELISA kit AP003 CRBS (EnviroLogix, Portland) were used to quantify the Cry1Ab/1Ac protein. Approximately 20 mg of fresh sample from leaf and stem (at the tillering/heading/filling stages), endosperm and brown rice (at the mature stage) was collected and ground into powder, which was then suspended in extraction buffer according to the proportion of 20 mg powder/500 μL extraction buffer and diluted to an appropriate concentration using dilution buffer. The dilution fold was 200 for the leaf tissue of the positive controls (T51-1 and Zheyou3) and lines LB/KEY and KEY/LB; 50 for the stem tissue of lines LB/KEY and KEY/LB and the positive controls and for the endosperm and brown rice of the positive controls; and zero for the negative controls and the endosperm and brown rice of the LB/KEY and KEY/LB lines. The enzyme-linking reaction was performed following the manufacturer’s instructions. The optical density values of the samples were measured at a 450-nm wavelength using a multi-mode microplate reader (Synergy H1, USA), and the values were used to calculate the content of Cry1Ab/1Ac protein in the samples.

### Field tests for insect resistance and agronomic performance

All the transgenic lines and the controls (wild-type Nipponbare and MH63) for insect resistance and agronomic trait and yield evaluation were planted in the containment field of the experimental farm of Zhejiang University in Hangzhou, China, in 2014 and 2015. In the preliminary test, carried out in 2014, the seeds were sown in a seedling bed in early June, and the seedlings were transplanted to the experimental fields approximately 25 days later. The field layout followed a randomized complete block design with three replications. Each plot consisted of 10 plants in one row with a distance of 20.0 cm between plants within a row and 40.0 cm between rows. The experimental plots were bordered by three rows of non-transgenic Nipponbare or MH63 rice plants. However, in the verification test carried out in 2015, each plot consisted of 24 plants in two rows with distances of 20.0 cm (26.7 cm for MH63) between plants within a row and 15.0 cm (20.0 cm for MH63) between rows and 40.0 cm between plots. All other conditions were the same. No chemical insecticides targeted against lepidopteran pests were applied throughout the growth period. Therefore, natural infestations of leaffolders were obtained. The reaction of the plants to the natural infestation of leaffolders was scored five to seven days after peak damage appeared. Leaves with visible scrapes and tillers with visibly scraped leaves were scored as damaged leaves and tillers, respectively. Both natural and manual infestations of striped stem borers were used. For manual infestations under field conditions, each rice plant was infested with 80 first-instar larvae of striped stem borers at both the late maximum tillering stage and late booting stage. Damage symptoms were checked 7 to 15 days after infestation. Dead hearts and white heads caused by striped stem borers were counted together to calculate the infection rate. For manual infestation under laboratory conditions, the fresh stem and flag and second-leaf sections were collected at the booting stage. Two stem sections in a tube and four leaf sections in a petri dish were tested for each plant. Three plants were tested for each line. The infestation dose was 20 first-instar (second-instar for plants with the MH63 background) larvae for stem sections and 15 for leaf sections per plant. Damage symptoms were checked 6 days after infestation.

To test the agronomic performance of the transgenic plants in the field, the plot was designed and used as above, except that chemical pesticides were applied throughout the growth period for crop protection. At maturity, the plant height was measured in the field, and all plants from each plot (15 individual plants per line were divided into three repetitions) were collected to measure the panicle length, number of panicles per plant, number of grains per panicle, seed-set rate, 1000-grain weight, and yield per plant.

### Statistical analysis

The data in each table was analysed by the Dunnett t test using the SPSS 22.0 software package, respectively. Statistically significant results at P < 0.01 and P < 0.05 are indicated in the tables.

## Electronic supplementary material

Supplementary Information
