# Supplementary material for: Application of Cre-lox gene switch to limit the Cry expression in rice green tissues
Source: Sci Rep. 2017 Nov 6;7:14505. doi: 10.1038/s41598-017-14679-0 (PMC5673937; doi:10.1038/s41598-017-14679-0)
Supplement: Supplementary file 1 — Supplementary Information [file 41598_2017_14679_MOESM1_ESM.pdf]

Supplementary information

Application of Cre-lox gene switch to limit the Cry expression in rice green tissues

Hao Chen[1, §], Ju Luo[2, §], Peng Zheng[1], Xiaobo Zhang[1], Cuicui Zhang[1], Xinyuan Li[1], Mugui Wang[1], Yuqing Huang[1], Xuejiao Liu[1], Mehmood Jan[1], Yujun Liu[1], Peisong Hu[2,*], Jumin Tu[1,*]

[§] These authors contributed equally to this work.

[*] Corresponding authors

The following supplementary information is available for this article:

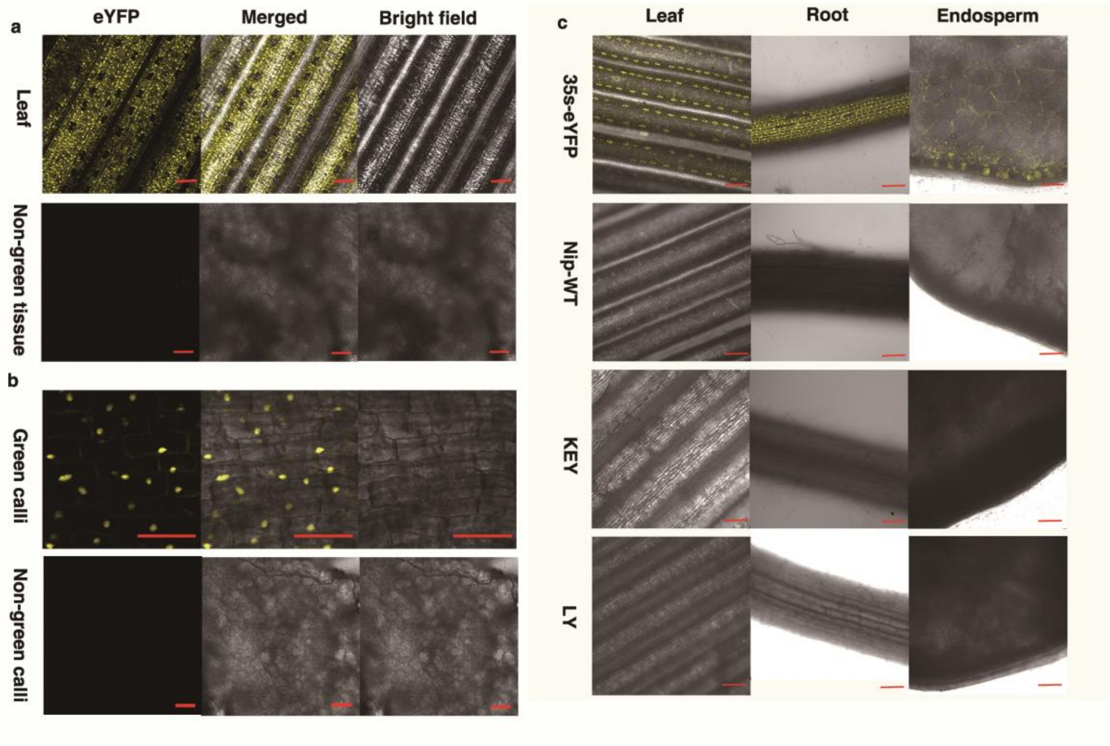

Supplementary Figure S1. Detection functions of the rice green tissue-specific *rbcS* promoter and nuclear import effect detection of *Arabidopsis* KRP2 in rice. (a), *pOsrbcS*::*eYFP* control vector expressed in the cells of rice seedling leaves. As shown in the figure above, the eYFP signal was detectable in the green leaf tissue, bar=100 µm; *pOsrbcS*::*eYFP* control vector not expressed in the subcultured non-green calli and eYFP signal was undetectable in the cells, bar=50 µm; **(b)**, *pOsrbcS*::*Krp2*::*eYFP* vector expressed in the regenerated green calli and showed eYFP singal in the nuclei, while *pOsrbcS*::*Krp2*::*eYFP* not expressed and no eYFP signal showed in the subcultured non-green calli, bar=50 µm. **(c)**, The parental KEY and LY lines showed no eYFP signal in the leaves, roots and seeds as negative control wild-type Nipponbare, 35S-eYFP line used as the positive control, bar = 100 µm.

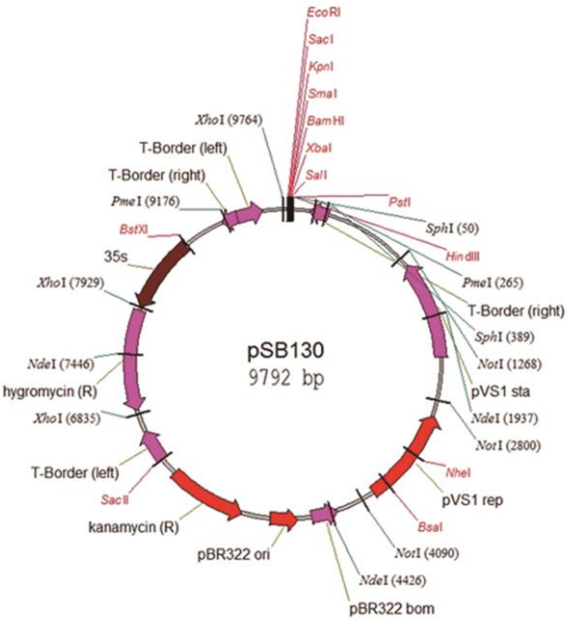

Supplementary Figure S2. Map of the vector pSB130. pSB130 is a standard binary vector, which contains two T-DNA region, the multiple cloning sites (MCS) is used to load the target fragments in T-DNA1, while the *hygromycin B phosphotransferase* (*HPT*) gene in T-DNA2 functioned as a transformation selectable marker.

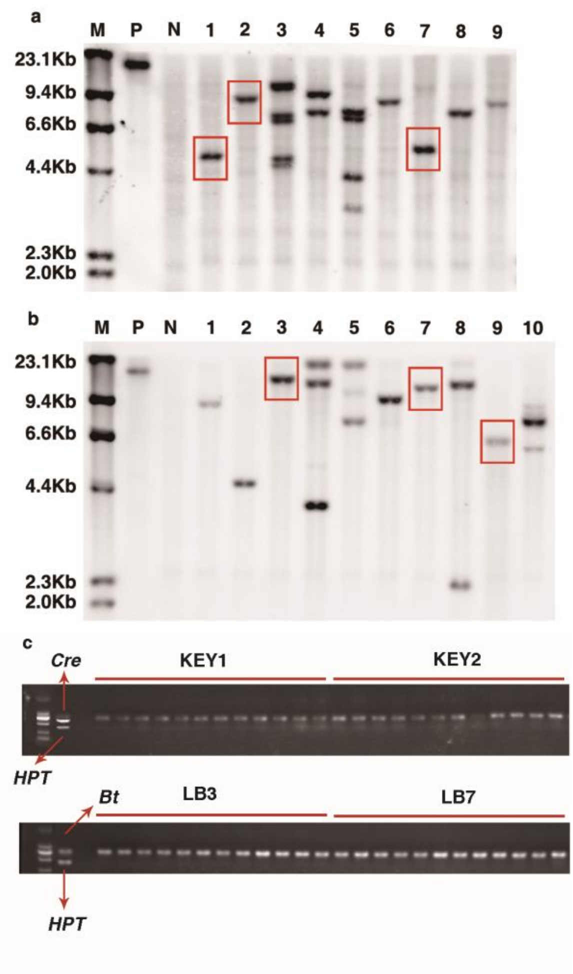

Supplementary Figure S3. ***Southern*** **blot analysis and** ***HPT*****-free lines selection of the parental KEY and LB lines.** The DNA samples extracted from the parental KEY and LB lines were digested with *Hin*dIII and hybridized with DIG probes, which were prepared from a PCR-amplified fragment of *Cre* and *Cry1Ab/1Ac* using DIG DNA Labelling Mix (Roche, Switzerland). M: DNA marker; P: Plasmid of pKEY or pLB; NT: Non-transgenic wild-type Nipponbare; lanes 1 to 9 or 10: independent transformants. **(a)**, Southern blot analysis of the KEY lines. Lanes 1, 2 and 7 indicate the KEY1, KEY2 and KEY14 lines, each with a single copy of KRP2-tagged *Cre* recombinase gene, respectively. **(b)**, Southern blot analysis of the LB lines. Lanes 3, 7 and 9 indicate the LB3, LB7 and LB9 lines, each with a single copy of the *NosT*-blocked *Cry1Ab/1Ac* insecticidal gene, respectively. **(c)**, PCR analysis of the *HPT*-free lines in the parental lines of KEY1, KEY2, LB3 and LB7.

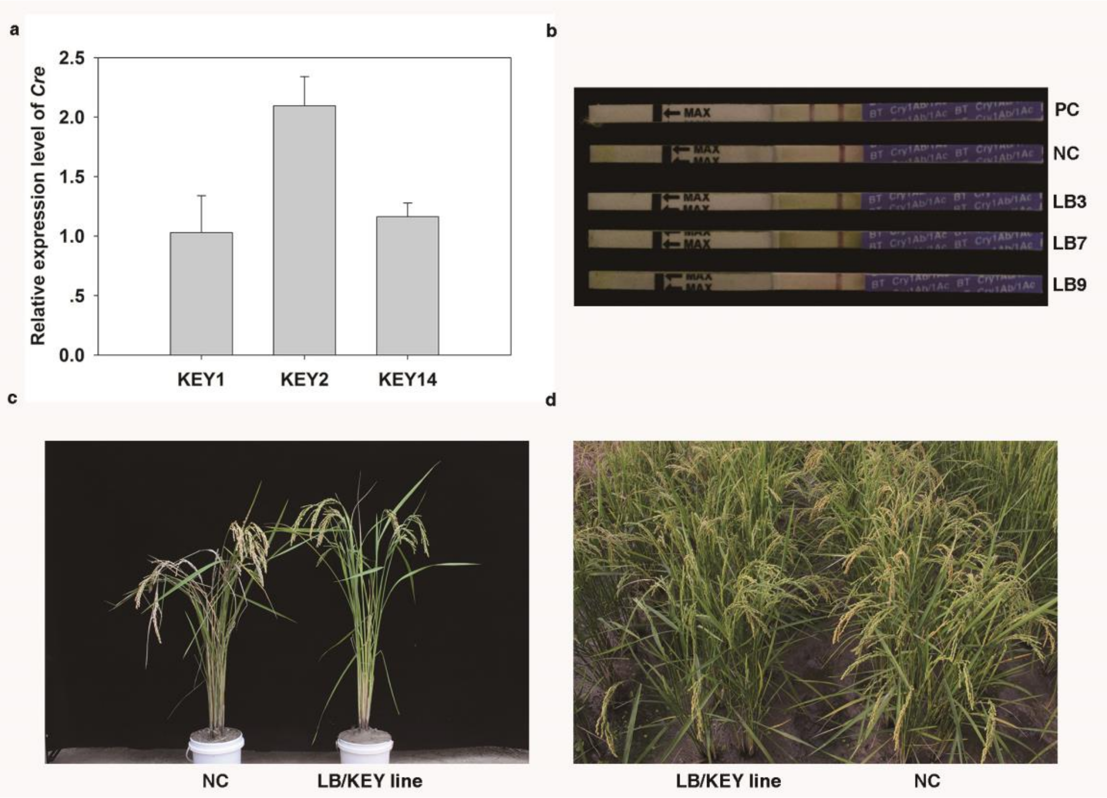

Supplementary Figure S4. Expression level of *Cre* in KEY lines, Bt protein detection in LB lines and the typical phenotype of LB/KEY hybrid line against striped stem borers and leaffolders. (a), Relative expression level of *Cre* in the leaves of KEY1, KEY2 and KEY14 lines, error bar meant SD. **(b)**, The Bt protein detection of the leaves of LB3, LB7 and LB9 lines using the Bt protein test strips, all lines didn't contain Bt protein, the T51-1 line and wild-type Nipponbare used as positive control (PC) and negative control (NC), respectively. **(c)**, The typical performance of LB/KEY lines against the manual infestation of striped stem borers in the field condition in 2014. **(d)**, The typical performance of LB/KEY lines against the natural infestation of leaffolders in the field condition in 2015, wild-type Nipponbare was used as negative control (NC).

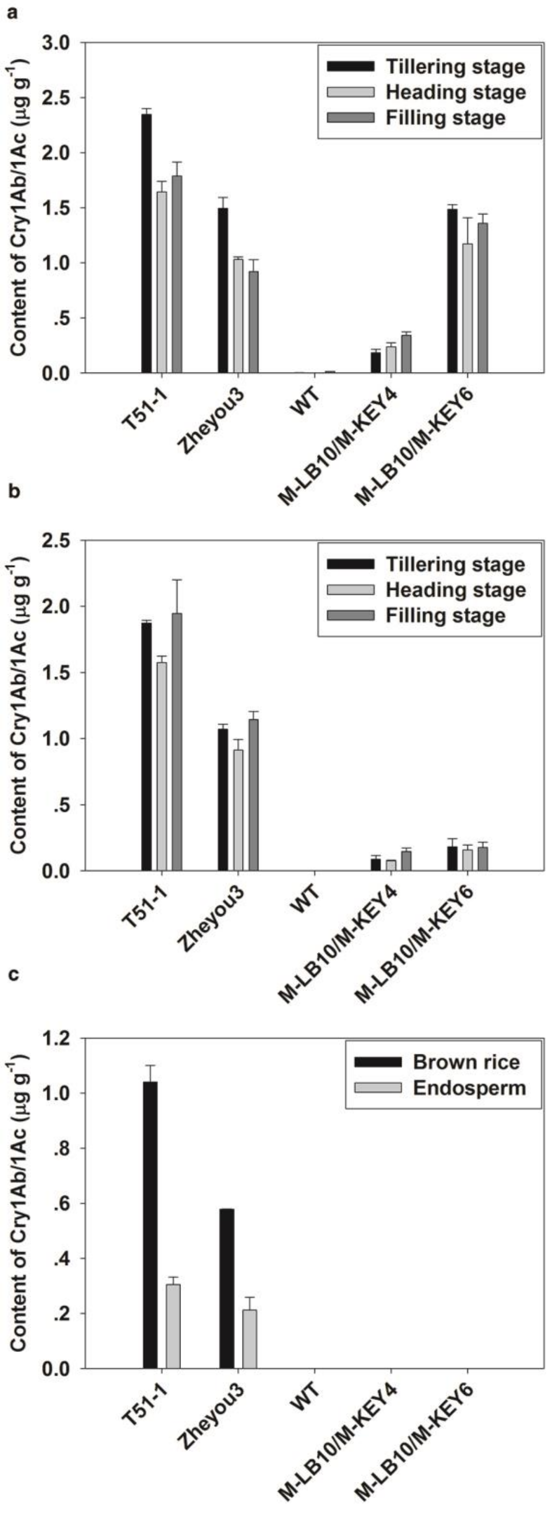

Supplementary Figure S5. The contents of Cry1Ab/1Ac protein in the M-LB/M-KEY lines of MH63 background. (a), **(b)**, **(c)**, The contents of Cry1Ab/1Ac protein in the leaves, stems, and seeds of the M-LB/M-KEY lines in 2015. Error bars indicate SD based on three biological replications and two technological replications. T51-1, Zheyou 3 were used as positive controls, wild-type MH63 was used as negative control.

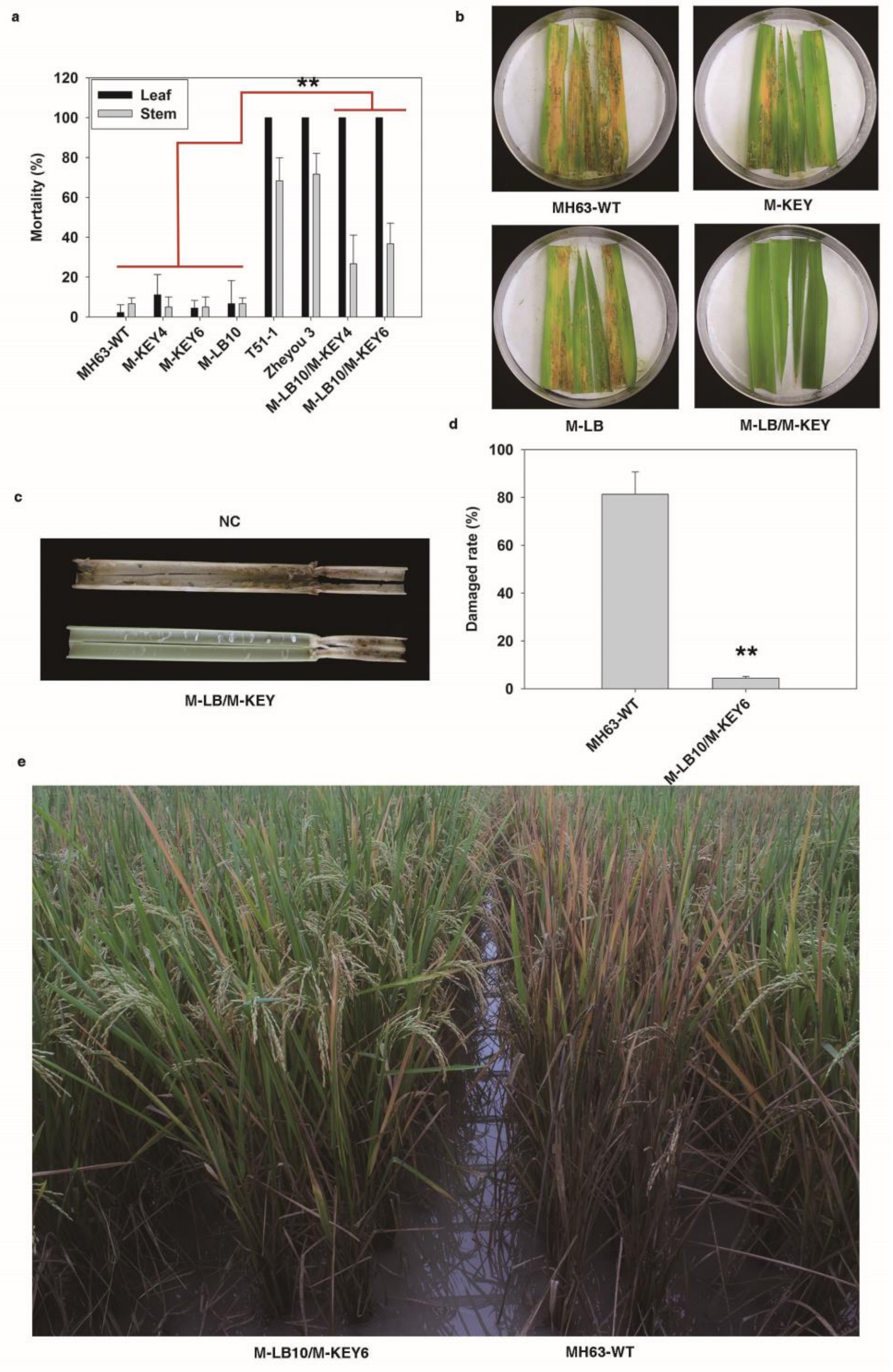

Supplementary Figure S6. R**esistance reactions of the hybrid M-LB/M-KEY lines against manual infestation of striped stem borers. (a)**, The mortality of larvae of leaf and stem sections from different tested lines against the manual infestation of striped stem borers under laboratory conditions, each line was tested for three replications. **(b)**, **(c)**, Typical resistance reactions of leaf and stem sections from different tested lines against the manual infestation of striped stem borers under laboratory conditions, respectively. **(d)**, Insect resistance evaluation of the hybrid M-LB10/M-KEY6 in 2015. The wild-type MH63 was used as a negative control. Each line was tested for three replications, and each replication contained 24 individual plants. **(e)**, The field performance of M-LB10/M-KEY6 against the manual infestation of striped stem borers. The wild-type MH63 was used as a negative control.

Table S1. Agronomic traits of the LB/KEY and KEY/LB hybrid lines, parental KEY and LB lines and wild-type Nipponbare under field conditions (2014, Hangzhou, China). All data were measured from 5 randomly sampled plants per test material per replication (3 replications) and analysed by the Dunnett t test using the SPSS 22.0 software package. Values were given as the mean ±standard deviation (SD). Nip-WT as the control and all of the other lines were compared with it. * and ** meant significantly different from the control in each year at $P < 0.05$ and $P < 0.01$, respectively.

Line	Plant height (cm)	Panicles per plant	Panicle length (cm)	Grains per panicle	Grains per plant	Seed-set rate (%)	1000-grain weight (g)	Yield per plant (g)
Nip-WT	93.40±0.89	11.27±0.42	18.68±0.44	90.53±3.64	1019.33±32.46	83.16±1.07	25.81±0.08	26.29±0.76
KEY1	94.43±0.86	9.87±0.12*	18.83±0.53	94.39±6.14	931.80±71.87	82.42±1.05	25.73±0.12	23.97±1.95
KEY2	93.77±0.12	10.93±0.95	18.76±0.28	91.21±5.78	1000.47±148.39	78.16±3.44**	25.74±0.17	25.72±3.95
KEY14	85.87±0.50**	13.93±1.29**	16.51±0.32**	63.79±2.69**	889.80±103.79	86.71±1.71*	26.15±0.18	23.30±2.60
LB3	94.90±0.70	10.20±0.60	18.85±0.17	95.04±4.42	969.73±78.41	82.47±1.44	25.52±0.42	24.73±2.03
LB7	94.47±1.33	12.73±0.95**	18.14±0.29*	91.37±4.16	1164.07±113.24*	75.28±2.70**	25.70±0.27	29.95±3.14
LB9	85.13±1.21**	13.07±0.31**	16.71±0.28**	61.03±3.11**	798.13±59.55**	82.48±1.38	26.09±0.18	20.80±1.51**
LB3/KEY1	95.57±1.26*	11.07±0.61	18.61±0.37	87.89±10.02	971.33±108.47	76.89±4.35**	26.03±0.20	25.33±2.99
LB7/KEY1	99.23±2.79**	10.60±0.40	18.90±0.07	105.13±1.25**	1114.67±52.82	85.57±0.97	26.07±0.21	29.07±1.21
LB9/KEY1	88.40±1.11**	10.80±0.60	18.56±0.19	99.12±10.65	1069.80±121.84	82.48±3.13	26.08±0.18	27.88±3.29
LB3/KEY2	94.73±1.21	11.00±0.20	19.14±0.23	101.57±1.10*	1117.27±26.83	84.62±1.35	25.91±0.67	28.92±1.44
LB7/KEY2	92.63±1.25	11.13±0.50	18.29±0.35	96.48±7.28	1071.67±31.70	84.43±1.54	26.22±0.24	28.13±1.11
LB9/KEY2	87.80±0.72**	10.80±0.35	19.10±0.08	103.27±6.23**	1116.33±95.70	85.59±1.91	25.97±0.13	28.95±2.33
LB3/KEY14	89.10±0.52**	11.33±0.50	17.87±0.20	83.26±3.77	942.67±28.21	90.76±2.38**	26.16±0.29	24.65±0.90
LB7/KEY14	92.47±1.06	11.60±1.25	18.79±0.32	87.69±8.72	1017.20±143.39	85.10±0.47	26.21±0.19	26.66±3.88
LB9/KEY14	85.87±0.50**	11.67±0.64	18.20±0.03	83.21±1.09	970.33±40.41	86.22±0.85	25.78±0.15	25.02±1.20

KEY1/LB3	95.47±1.72*	11.40±0.00	18.70±0.12	93.84±0.99	1069.80±11.26	82.57±0.99	26.08±0.14	27.89±0.31
KEY2/LB3	93.57±0.95	10.93±0.50	18.56±0.28	93.15±2.38	1017.87±35.72	82.25±0.48	25.93±0.63	26.41±0.68
KEY14/LB3	88.73±0.31**	11.67±0.42	18.22±0.24	87.41±4.27	1019.93±66.00	89.98±0.57**	26.07±0.25	26.59±1.88

Table S2. Agronomic traits of the LB/KEY and KEY/LB hybrid lines, parental KEY and LB lines and wild-type Nipponbare under field conditions (2015, Hangzhou, China). All data were measured from 5 randomly sampled plants per test material per replication (3 replications) and analysed by the Dunnett t test using the SPSS 22.0 software package. Values were given as the mean ±standard deviation (SD). Nip-WT as the control and all of the other lines were compared with it. * and ** meant significantly different from the control in each year at $P < 0.05$ and $P < 0.01$, respectively.

Lines	Plant height (cm)	Panicles per plant	Panicle length (cm)	Grains per panicle	Grains per plant	Seed-set rate（%）	1000-grain weight (g)	Yield per plant(g)
Nip-WT	93.67±0.40	10.87±0.81	16.96±0.46	90.72±5.74	985.13±87.38	93.05±2.20	25.80±0.10	25.42±2.28
KEY2	93.80±0.17	11.00±0.69	17.49±0.31	85.98±3.20	945.13±56.12	91.91±1.20	25.73±0.13	24.32±1.51
LB3	94.50±0.17	11.53±1.10	17.75±0.41	94.21±5.41	1083.00±55.61	91.82±0.50	25.51±0.17	27.62±1.26
LB7	94.33±0.95	12.53±1.33	15.11±0.08**	72.84±3.16**	910.60±66.69	85.56±1.92**	25.63±0.15	23.33±1.86
LB3/KEY2	94.47±0.76	12.00±1.04	17.46±0.74	89.73±10.22	1073.27±117.03	94.70±0.72	25.83±0.35	27.68±2.64
LB7/KEY2	93.90±0.66	14.87±1.40**	16.43±0.23	80.85±2.87*	1203.13±135.31**	89.43±1.09**	25.77±0.06	30.94±3.52**
KEY2/LB3	94.60±0.72	11.13±0.42	17.61±0.61	86.65±3.99	963.93±33.61	90.98±0.98	25.62±0.02	24.70±0.87

Table S3. Resistance reaction of M-LB/M-KEY hybrid lines, the parental M-KEY, M-LB lines and wild-type MH63 control against natural infestation of striped stem borers and leaffolders under field conditions (2015, Hangzhou, China). All dada were measured from 10 randomly sampled plants per test material per replication (3 replications) and analysed by the Dunnett t test using the SPSS 22.0 software package. Values are given as the mean ± standard deviation (SD). MH63-WT (wild-type MH63) as the negative control and all of the others lines were compared with it. * and ** meant significantly different from the control at P < 0.05 and P < 0.01, respectively.

Lines	Damaged by striped stem borers（%）	Damaged by leaffolders	
		Tillers damaged per plant (%)	Leaves damaged per plant
MH63-WT	13.77±2.57	54.83±3.11	12.07±0.71
M-KEY4	13.89±1.69	59.70±3.66*	12.60±0.82
M-KEY6	13.09±1.19	56.12±1.99	11.67±0.06
M-LB10	13.24±1.39	56.31±2.25	12.27±0.47
M-LB10/M-KEY4	1.67±0.67**	0.84±0.73**	0.17±0.15**
M-LB10/M-KEY6	0.63±0.05**	0.43±0.37**	0.07±0.06**

Table S4. Agronomic traits of the hybrid M-LB10/M-KEY6 line and wild-type MH63 under field conditions (2015, Hangzhou, China). All data were measured from 5 randomly sampled plants per test material per replication (3 replications) and analysed by the Dunnett t test using the SPSS 22.0 software package. Values were given as the mean ±standard deviation (SD). MH63-WT as the control and the hybrid M-LB10/M-KEY6 line was compared with it. * meant significantly different from the control at $P < 0.05$.

Lines	Plant height (cm)	Panicles per plant	Panicle length (cm)	Grains per panicle	Grains per plant	Seed-set rate (%)	1000-grain weight (g)	Yield per plant(g)
MH63-WT	100.40±0.44	13.27±0.70	23.23±0.47	106.36±0.66	1402.67±77.12	79.04±6.58	27.36±0.33	38.38±2.10
M-LB10/M-KEY6	104.77±2.95*	14.93±2.27	23.91±0.84	108.62±5.02*	1727.33±100.60	79.99±1.10*	27.60±0.35	47.66±2.38

Table S5. Primers used in this study.

Primer	Sequences (from 5' to 3')
For positive tests of transgenic plants	
Cre-F	AACGAGTGATGAGGTTCGCA
Cre-R	ACCCGGCAAAACAGGTAGTT
eYFP-F	CTACCCCGACCACATGAAGC
eYFP-R	CGCTTCTCGTTGGGGTCTTT
Bt-F	GGCCATACAACTGCTTGAGT
Bt-R	GCGTTTCCCATAGTTCCATA
HPT-F	TTGGGAATCCCCGAACATCG
HPT-R	TGACCTATTGCATCTCCCGC
For DNA recombination validation	
eYFP-switch-F	GGGGAAAAGGGCACTATGGTT
eYFP-switch-R	GTGCAGATGAACTTCAGGGTC
For Southern blot	
Cre-SB-F	ATGTCCAATTTACTGACCGT
Cre-SB-R	GCTTCAAAAATCCCTTCCAG
Bt-SB-F	AGGCTGATTGGAAACTACACCGAC
Bt-SB-R	ACAGCGGATGGCAAGTTAGAAGAG
For qRT-PCR	
Actin-qRT-PCR-F	TGGCATCTCTCAGCACATTCC
Actin-qRT-PCR-R	TGCACAATGGATGGGCCAGA
eYFP-qRT-PCR-F	TTCAAGTCCGCCATGCCCGAA
eYFP-qRT-PCR-R	TTGCCGTCCTCCTTGAAGTCG
Cre-qRT-PCR-F	ACCCTGTTACGTATAGCCGAA
Cre-qRT-PCR-R	GCTAAGTGCCTTCTCTACACC
